# Investigations of the Influence of Nano-Admixtures on Early Hydration and Selected Properties of Calcium Aluminate Cement Paste

**DOI:** 10.3390/ma15144958

**Published:** 2022-07-16

**Authors:** Renata Boris, Iwona Wilińska, Barbara Pacewska, Valentin Antonovič

**Affiliations:** 1 Laboratory of Composite Materials, Institute of Building Materials, Vilnius Gediminas Technical University, 08217 Vilnius, Lithuania; valentin.antonovic@vilniustech.lt; 2 Faculty of Civil Engineering, Mechanics and Petrochemistry, Warsaw University of Technology, 09-400 Płock, Poland; iwona.wilinska@pw.edu.pl (I.W.); barbara.pacewska@pw.edu.pl (B.P.)

**Keywords:** calcium aluminate cement (CAC), hydration, nano admixtures, calorimetry, microstructure

## Abstract

In this work, the hydration of calcium aluminate cement (CAC, Al_2_O_3_ ≥ 70%) paste with nano admixtures (0, 0.05%, 0.1% and 0.2%) of nano-silica (NS) and carbon nano-cones (NC) when W/CAC = 0.35 was investigated. The methods of calorimetry, thermal analysis, X-ray diffraction (XRD), IR spectroscopy, and scanning electron microscopy (SEM) were used. In addition, the physical and mechanical properties of hardened cement pastes were determined after 3 days of hardening. NS was found to shorten the induction period of CAC hydration and accelerate the time of the secondary heat release effect, especially in the specimens with the highest NS content. The incorporation of NC (up to 0.2%) slows down the hydration process. After 3 days of hydration, the formation of hydration products, such as C_2_AH_8_, CAH_10_, C_3_AH_6_ and AH_3_ hydrates, was observed in CAC pastes, however, the quantitative compositions were different depending on the kind of nano admixture and its amount. SEM results obtained show differences in the effect of NS and NC on the formation of the structure of cement paste during its hardening. Significant changes in CAC paste microstructure were caused by the addition of NS and NC admixtures. Compressive strength was found to increase with the increase of NS and the optimal NS content was found to be 0.10 wt.%. The modification of the cement paste with an NS admixture results in a higher amount of hydrates, lower total porosity, and a higher amount of the smallest pores in the microstructure of the sample. NS and NC influence the hydration behaviour of CAC in different ways, which causes characteristic changes in the microstructure and properties of hardened samples.

## 1. Introduction

Nowadays, nanotechnology plays an important role in the development of cement-based materials. This applies both to composites made of common Portland cement as well as special cements. Calcium aluminate cement (CAC), as a kind of special high-performance cement, is widely used in refractory industries, industrial floors, and repair of constructions owing to its excellent combination of properties, including high early strength and fast hardening rate [1]. The hardening process of CAC compared to Portland cement goes very quickly and binds the components of refractory castable to a strong system during thermal treatment.

Necessary components for the new generation of materials are nanoparticle additives and admixtures. Research on the use of nanomaterials in cement and concrete has a great interest in the following aspects: obtaining more unique properties of cementitious materials, more efficient cement hydration, improving the durability of materials (better resistance to high temperatures), achieving ultra-high compressive strength, and controlling crack formation [2,3].

Different mechanisms of acting of nano additives in cement composite contribute to the improvement of mechanical and physical properties of hardened cementitious material [4,5]. An analysis of the literature shows that nano additives can act as crystallization centres [5,6], hydration accelerators [5,7,8], or retarders [3,7,8]. Acting as filler, nano particles can make the microstructure of cement composite denser and more homogenous. In the presence of nano particles, hydration products may be fine, preventing them from growing [4,5,7].

Papers relating to the influence of different nano particles on the properties and hydration processes of Portland cement composites can be found [5,9,10,11,12,13,14,15]. However, there is less research work on the similar influence of nano particles on CAC. Knowledge about the development of calcium aluminate microstructures in the presence of nano components as well as their impact on CAC hydration processes is necessary to understand the development of physical and chemical properties of the final hardened material.

Nano-silica is the most widely used in Portland cement-based materials [14]. There is also interest in the use of nano-silica in compositions with CAC.

The authors of [4] found that nano-SiO_2_, used in an amount of 1%, improved 7 day compressive strength and the microstructure of monocalcium aluminate cement paste. Further addition of nano-SiO_2_ resulted in a slight reduction of mechanical properties. Wang et al. [16] investigated the hydration process in cement pastes containing a higher amount of colloidal silica. They concluded that silica nanoparticles promote early and inhibit later hydration periods. The latter effect occurs because nano-silica influences the microstructure of hardened pastes, making it denser compared to CAC pastes without the admixture. The investigations of other researchers show that in the presence of nano-silica, hydrated calcium aluminosilicate products, such as C_2_ASH_8_, can be formed (abbreviations used in cement chemistry: C—CaO, A—Al_2_O_3_, S—SiO_2_, H—H_2_O) and the conversion of hexagonal to regular aluminate hydrates is inhibited [6,17,18,19].

Nano carbon materials used in small amounts (usually below 0.5 wt.%) can be introduced to Portland cement composites to improve their properties [20]; however, such admixtures are rarely found in the case of CAC cements. The results obtained in a PhD thesis [21] show the influence of nano graphene (used in the amount of 0.07%, 0.14% and 0.28%) on physical and chemical properties of CAC paste. The author of [21] found that nano graphene accelerates the cement hydration process and changes the amount of hydration products. According to the results of ultrasonic pulse velocity, a denser microstructure was formed in a composition with nano graphene in the early CAC hydration stage (24 h). The work showed that the compressive strength increases from 5% to 19%, depending on the thermal treatment temperature, in the refractory castable with 0.07% amount of nano graphene.

Furthermore, until now, no studies have been performed yet with carbon nano-cones in CAC based composites. However, a review [22] reports that nanosheets (MXene) have an effect in cement-based materials for photocatalysis on mechanical properties and promote industrial upgrading of new building materials.

The use of other different nano materials in CAC composites, e.g., nano-γAl_2_O_3_ [23], nano-TiO_2_ [7], nanocelluloses [24], carbon nanofiber [25] and carbon nanotubes, has also been studied [2].

In the case of CAC refractory castable, calcium aluminate phases developed during the hardening process undergo transformation at high temperatures and form strong ceramic bonds [26]. The aim of using nanotechnology in refractory materials is to improve some of their properties, such as compressive strength, bond strength between aggregate and cement paste, thermal durability, resistance to abrasion, and chemical corrosion. The properties depend on the structure of the material, thus the possibility to control the kind and size of hydrates formed during CAC hydration is very important. The formation of nanoscale amorphous hydrates and retardation of the development of large crystals are desirable [27].

In order to understand the role of active components of the refractory binder in the development of the properties of concrete, it is necessary to investigate the physicochemical processes taking place in the material at various stages of its preparation, including hydration and thermal treatment. Then, based on the obtained results, it is possible to determine the relationship between these processes and the properties of the final composite. It should be noted that CAC hydration products strictly depend on temperature. Thus, the degree of CAC hydration, the kinds of hydrated aluminate phases and the development of microstructure influence mechanical properties of concrete [20,28].

There is still a need for research on the influence of nano admixtures on CAC hydration and the properties of refractory materials.

The aim of this work was to determine the effect of admixtures, such as nano-silica (NS) and carbon nano-cones (NC), on early hydration of CAC and selected properties of the modified cement paste. According to our knowledge, the influence of nano-silica has been studied to a small extent, while carbon nano-cones have not been used so far as admixtures to modify the properties of composites made of CAC. Different mechanisms of interaction of nano particles in cement binder may be considered, such as: the filling effect, the nucleation effect, and the chemical effect. NC belong to the group of nano-carbon materials, however, due to the shape and size of the grains, it can be expected that their interaction with the cement system will be different from that of nanotubes, nano-fibers and other nano-carbon materials previously described in the literature. In addition, the effect of NC will be different from NS not only due to the kind of grains but also due to the chemical composition. Carbon nano-cones in the cement system are expected to mainly play the role of solid nanofillers, while nano-silica also shows chemical activity. Therefore, the research described in this paper is justified in order to broaden knowledge on the action of nano-silica in CAC and compare it with the effect of chemically inactive material not previously used in admixtures for CAC-based binders. The mechanism of early CAC hydration was investigated by calorimetry, SEM microscopy, X-ray diffraction, thermal analysis, and infrared spectroscopy. Moreover, the impact of NS and NC on the flowability as well as porosity, density, and compressive strength of the hardened cement paste was also investigated. This work is the first step in comparative investigations of the possibilities of using these admixtures in refractory castable.

## 2. Materials and Research Methods

Testing was performed with a CAC Gorkal 70 (G70), produced by Górka Company in Trzebinia (Poland). The chemical composition and main phases are shown in Table 1. The minerals monocalcium aluminate (CA) and calcium dialuminate (CA_2_) are predominant. The phases α-Al_2_O_3_ and C_12_A_7_ were also identified and existed in small amounts in CAC. Other properties of G70: specific surface area (Blaine fineness) 400–450 m^2^/g, pH 11–11.5, and bulk density 1100 kg/m^3^ [29].

Nano-silica (NS) properties: purity 99.8%; particle surface area: 202 m^2^/g; pH (40 g/L) 4.0; relative density: 2.2 g/cm^3^; and size of grains: 10–30 nm (TDS of Sigma-Aldrich Chemie GmbH, Merck, Taufkirchen, Germany). The pozzolanic activity of NS is 1695 mg/g (according to NF P18-513 Chapelle test) [15]. Regarding the grains shape and size, NS can be classified as a three-dimensional (spatial) nano material.

**Figure 1:**
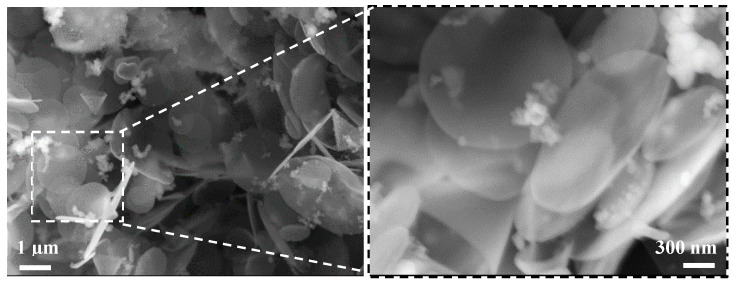
Micrographs of NC obtained by SEM/SE.

Carbon nano-cones (NC) are nearly perfect carbon sheets (open circles) from the company n-Tec (Norway). Properties: diameter 800–3000 nm; sheet thickness: 20–50 nm; chemical composition (weight, %): C—98.47; H—0.77; N—0.54, S—0.60; thermal stability: 500 °C. The image of the NC microstructure is presented in Figure 1. The grains of NC are larger compared to NS; they can be classified as two-dimensional (planar surface) nano materials.

Cement paste samples produced from the following raw materials were tested: G70 (G0) and mixtures of G70 with the addition of NS (GNS1, GNS2, GNS3) and NC (GNC1, GNC2, GNC3). Distilled water was used for making the samples. The water to cement ratio (W/C) was 0.35. The compositions with and without nano admixtures used for the tests described in this paper are presented in Table 2.

The mixing procedure for the cement paste preparation was as follows. First, the nanomaterial particles were dispersed in water for 5 min at 400 W and 22 kHz (in an ultrasonic disperser UZDN-2 T). Next, the resulting suspension was cooled to temperature 20 ± 1 °C; and then the prepared suspension was poured into the CAC. Finally, the mixtures were blended in the Hobart mixer for 5 min. The control cement paste without admixtures was prepared using the same mixing procedure. After the mixing, the cement paste was poured into polyethylene bags. To avoid self-heating, the pastes in all bags were evenly distributed to form a thin, 5 mm-thick layer. Polyethylene bags were tightly closed and put into the climatic test chamber 3401 RUMED. The samples were cured at the temperature of 20 °C. Prisms with dimensions 160 × 40 × 40 mm were moulded for the testing of density, compressive strength, and porosity of hardened cement pastes (Table 2). The samples were demoulded after 24 h and cured for three days before the testing according to the procedure described in LST EN ISO 1927-5:2013.

The amount of heat released during the samples’ hydration and the heat release rate are measured by the calorimeter TONICAL III (Toni Technik GmbH, Berlin, Germany). The mixtures (35 g of water and 100 g of a solid substance) were studied for 48 h at the operating temperature of 20 °C, and the heat evolution curves were registered.

The rheological properties were measured according to LT EN 1015-3:2007. Flow table tests of fresh cement pastes were carried out immediately after mixing. The ring mould (∅ 30 mm, h 50 mm) was placed centrally on the disc of the flow table and cement paste was introduced in two layers, each layer being compacted by at least 10 short strokes of the tamper to ensure filling of the mould. After 15 s, the mould was slowly raised vertically and after that, it was given 15 vertical impacts by raising the flow table. The flow value was measured by the mean diameter of a test sample (in two directions) of the fresh paste. Percentage values were calculated.

The microstructure analysis and the phase composition were tested immediately after 3 days of hardening.

The microstructure analysis was performed using a scanning electron microscope (SEM) JSM-7600F (JEOL, Tokyo, Japan). The analysis was done at an accelerating voltage of 4 or 10 kV, the mode of secondary electrons was used in image formation. Before the investigation, the surface of small pieces of specimens to be investigated was covered with a layer of electricity conducting material using a QUORUM Q150R ES (Quorum Technologies Ltd., Reutlingen, Germany) device. Small pieces of pastes were taken.

The X-ray diffraction (XRD) analysis was performed using a DRON-7 diffractometer (Bourevestnik, St. Petersburg, Russia) with Cu-Kα (λ = 0.1541837 nm) radiation. The following test parameters were used: 30 kV voltage; 12 mA current; 2θ diffraction angle range from 4° to 60° with increment of 0.02° measured each 2 s. Phases present were identified comparing the XRD diffractograms with standard diffraction patterns provided by the International Centre for Diffraction Data (ICDD). The internal standard anatase was used. Specimens were prepared using a mass ratio of 9:1 (sample:anatase). The amounts of compounds were valued according to the intensity of the main peaks. The height of the peaks of all diffractograms was adjusted so that the intensity of the main anatase peak (2θ = 25.28°) would be the same among all diffractograms. As such, the relative intensity of the other peaks is comparable.

Before TG/DTG/DTA and IR measurements, hydration was stopped by the use of acetone. The samples of hardened cement paste were immersed in acetone for 72 h and then dried in an electric oven at 50 °C for 72 h [15,30].

Investigation of hydration products with means of IR spectroscopy was conducted using a Genesis II FTIR spectrometer (Mattson, Madison, WI, USA), within the range of spectra: 4000–400 cm^−1^. The samples were prepared in the form of KBr pellets.

Thermal analysis (TG/DTG/DTA) was performed by the use of an STA 2500 Regulus (Netzsch, Germany) thermoanalyser. The tests were performed in the temperature range from 30 to 900 °C in a nitrogen atmosphere, the rate of heating was 10 °C/min, and the mass of the sample was 15–23 mg.

Three samples of each cement paste were tested for density and for compressive strength. The density of the samples after hardening was calculated according to the sample mass (accuracy 0.01 g) and volume determined by dimensions (accuracy 0.01 mm). The compressive strength was measured using a hydraulic testing machine ALPHA3-3000 S (Tinus Olsen, JAV) according to LST EN ISO 1927-6:2013.

The porosity of samples were investigated by mercury intrusion porosimetry (MIP) using a Quantachrome Poremaster 33/60 (Quantachrome Instruments, Boynton Beach, FL, USA) with a maximum pressure of 33,000 psi for pore size measurements ranging from 900 μm to 0.006 μm in diameter, and with two low-pressure stations plus one high-pressure station. The specimens after thermal treatment at the temperature of 105 ± 5 °C (for 72 h) were tested.

## 3. Results and Discussion

### 3.1. Calorimetry Measurements

Compositions of tested cement pastes used in the studies are presented in Table 2. Compositions with W/C = 0.35 were used, as this ratio ensures the formation of a denser and non-layered structure (close to the normal consistency).

**Figure 2:**
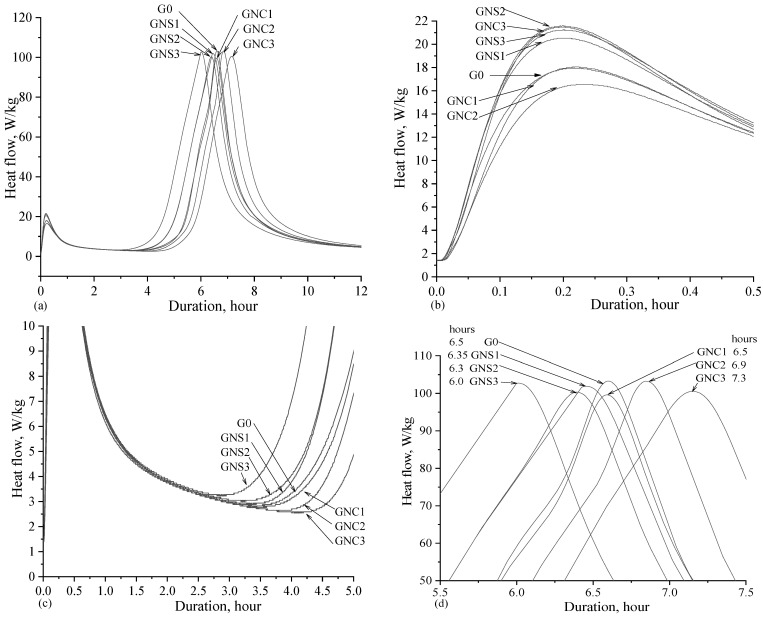
Heat flow curves of cement pastes with NS and NC admixtures (**a**–**d**).

Cement hydration periods and their categorization are described in detail in [7,29,31,32]. Therefore, the interpretation of the results from the calorimetric analysis is conducted based on this literature. The heat flow of cement paste was studied using calorimetry studies during 48 h of hydration and the results are shown in Figure 2. The exothermic processes of hydration reactions show the five typical stages in heat flow curves. The wetting/dissolution corresponds with the first peak, that is, initial hydration (Stage I; Figure 2b) on the surface of cement grains. After this stage, the induction period takes place. During the induction period, the heat release is almost inhibited, the concentration of ions in the solution increases until the maximum value is reached, and the nuclei of hydration products appear and grow. It can be seen that NS admixture in the cement paste shortens the induction period (Stage II; Figure 2c). Another effect was observed in the case of NC admixture, i.e., the extension of the induction period. Additional surface area provided by fine-grained NS could have enabled the formation of more nuclei of CAC hydration products. A similar conclusion was drawn by other authors [33] who investigated the influence of different ultrafine fillers on the rate of CAC hydration. The acceleration period (Stage III; Figure 2d) is the stage between the end of the induction period and the second exothermic peak in hydration heat release. It corresponds to cement hydration (massive precipitation of hydrated products, intensive release of heat). Performed calorimetry tests showed that a higher amount of NS admixture increased from 0.05% to 0.2% accelerated the appearance of the maximum peak of cement hydration of stage III in the calorimetry curve from 6.35 to 6.0 h in comparison to 6.5 h in the case of the reference sample. The effect of NC admixture (0.05% to 0.2%) on cement hydration is different: the increased amount of NC slows down the hydration and shifts the exothermic peak from 6.5 to 7.3 h. The acceleration of the crystallization of hydrates can be explained as follows: NS grains influence the formation of new crystallization centres, which promote the formation of hydration products and thus accelerate CAC hydration. Later, in the deceleration period, hydration processes slow down and less heat is released (Stage IV). The final period, the steady state (Stage V), is called the diffusion-limited reaction period.

**Figure 3:**
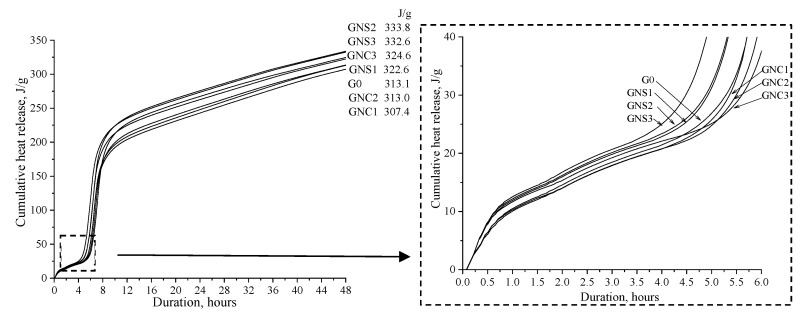
Cumulative heat release of cement pastes with NS and NC.

The cumulative heat release in the cement pastes containing NS and NC admixtures was measured up to 48 h (Figure 3). Compared to the control sample (G0), the highest amount of total heat released in the samples with NS (0.05%, 0.1% and 0.2%) and NC (0.2%) was observed. The most intense effect of nano admixtures was visible when 0.1% of NS was added (the gain of heat released after 48 h of hydration was more than 6% for the samples GNS2 and GNS3). The total heat released in the samples with NC was similar to the value registered for the reference cement paste. The addition of NC at 0.05% and 0.1% slightly reduced the cumulative heat released by ~2% compared to the control sample, with the exception of the sample containing 0.2% of NC, which showed an increase in heat released.

Thus, the results of calorimetric measurements show that the used admixtures affect the early hydration of CAC in various ways: NS accelerates the hydration while NC decelerate it slightly. The possible reason is that the addition of nano-silica promotes the formation of hydration products and releases more heat (Figure 2b and Figure 3). In addition, more nano-silica seem to speed up the hydration process of CAC and the heat release rate (Figure 2c,d). Cumulative heat release (Figure 3) increases when the admixture content of nano-silica is higher and decreases with the addition of NC.

NS has significantly smaller grains compared to NC and thus provides high amounts of nucleation centres, which accelerate cement hydration. The improvement of cement dissolution in the presence of NS should also be considered. On the other hand, it is possible that, at the early hydration stage, a part of NC is adsorbed on the grains of cement, similarly as in the case of using other carbon nanomaterials in Portland cement composite [21,34].

### 3.2. Flowability Properties of Cement Paste

**Figure 4:**
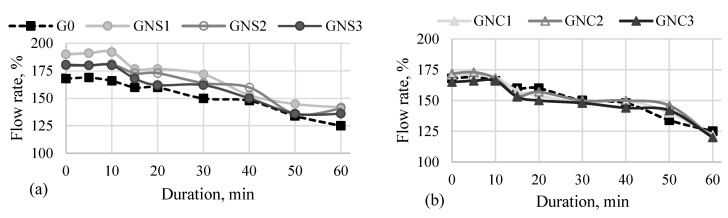
Flowability of cement pastes during 60 min. (**a**)—reference sample and sample with NS; (**b**)—reference sample and sample with NC.

The influence of NS and NC admixtures on the flowability of cement pastes during 60 min after adding water was tested. The results are presented in Figure 4.

The tests show that the nanoparticles used influence the change of the cement paste flow rate. It depends on the type of the admixture and the time passed after adding water. The test results showed that a low amount of nano-silica added to the cement in the form of ultrasonically dispersed suspension in water improved the flowability of the cement paste compared to the reference sample without nano admixtures. The highest positive effect on the cement paste workability was observed in the case of the lowest amount of nano-silica (GNS1). Larger amounts of NS reduce the flowability due to the high specific surface area of the admixture; however, the flow rate is still higher compared to the result for the reference. It can therefore be expected that in the presence of NS, the dissolution of cement components is improved, which contributes to the acceleration of hydration processes.

The effect of NC after adding water to the cement is different than that of NS. The level of flow rate is similar to the result for the reference paste, while in the case of the highest amount of NC (GNC3), the flowability is slightly reduced.

Analyzing the changes of the flow rate over time, it can be concluded that the flow rate of samples GNS1-3 gradually increases during the first 10 min, starting from the time of preparation of the cement paste. Meanwhile, in the case of GNC1-3 and G0, the flow rate increases within 5 min after the preparation of cement paste and then it starts constantly decreasing. A decrease in the flow rate is clearly observed in all paste samples 10 min after the preparation of the paste, (Figure 4). This time corresponds well with the time of the first effect of heat release (wetting) on the calorimetric curve (Figure 2a,b). The results show that the hardening starts after 10 min, but the intensity of the process depends on the kind of the admixture. In the case of flow curves registered for the NS-admixtured pastes, one can see that the flow rate drops significantly over the time of the test, while in the case of NC-admixtured pastes, the flow rate shows some stabilisation between 20 and 40 min, and then, after 50 min, it decreases. Thus, the results confirm that NS accelerates early hydration and hardening processes of cement paste while NC decelerate them slightly. The conclusion agrees with the results of cumulative heat released after 60 min of hydration (Figure 3): the total amount of heat released is higher for the pastes containing NS, while it is slightly lower than the reference for the pastes containing NC.

It should be noted that the more flowable cement paste prepared with NS was used for the investigation of the hydrates formed during the hardening of the paste as well as for the study of physical-mechanical properties (density, compressive strength and MIP) in parallel to the less flowable mortar prepared with NC. Flowability is one of the factors influencing the microstructure of cement pastes and the hydration products formed.

### 3.3. SEM Analysis

**Figure 5:**
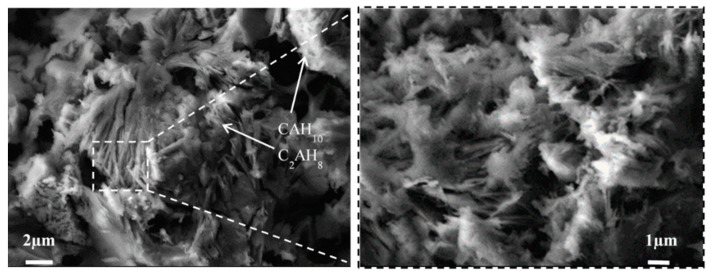
The microstructure images of control specimen G0 after 3 days of hardening.

**Figure 6:**
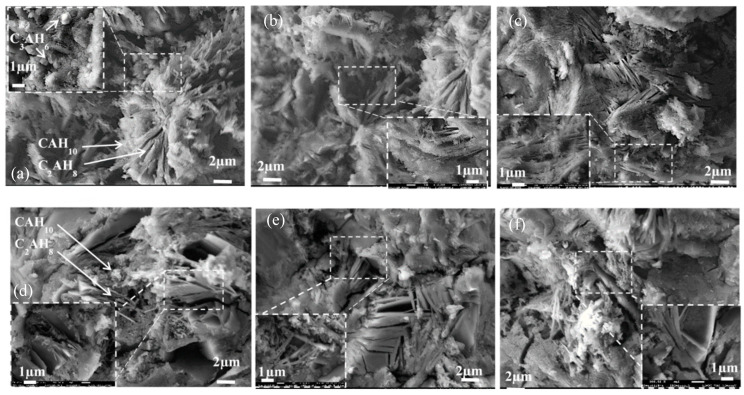
The microstructure images of samples after 3 days of hardening. (**a**)—GNS1; (**b**)—GNS2; (**c**)—GNS3; (**d)**—GNC1; (**e**)—GNC2; (**f**)—GNC3.

Split surfaces of small pieces of cement paste after 3 days of hardening were used to investigate the microstructure of control cement samples without admixtures (Figure 5) and to understand the impact of nano admixtures (NS and NC) (Figure 6). In general, CAH_10_ and C_2_AH_8_ are visible in the structure of hardened cement pastes. The SEM analysis showed that a more densified structure was formed in the hardened cement sample with NS (Figure 6a–c); however, it is more difficult to identify hydrates from these microstructure images. The admixture of NS is possibly more reactive [35] and the smallest hydrates which were formed in its presence are difficult to identify. In the samples with NC, separate plates of C_2_AH_8_ hydrates are visible and the NC admixture seems to repel the forming plates (Figure 6d–f). Other fine forms of hydrates are also visible, especially in the CNS1 sample, as shown in Figure 6a; they are probably small cubic hydrates of C_3_AH_6_.

The analysis of SEM images of hardened cement pastes revealed that in the samples with silica nanoparticles, the microstructure is more stable, denser, and the crystals are oriented in one direction. In the microstructure of the samples with NC, the distribution of crystals is more chaotic with gaps between crystal plates, but it seems that admixtures fill the gaps. In the structure of the samples with NS, a higher number of small pores can be observed (Figure 6b). The structure of the sample with NC is different; more large pores and gaps can be observed (Figure 6d–f).

The stated causes of changes in the flowability of cement pastes GNS1-3 over time were confirmed by SEM analysis showing that the microstructure of the sample surface was more densified (Figure 6a–c). The microstructure is less mechanically damaged during the splitting; fewer pores and cracks are visible in all SEM images (Figure 6a–c). We can judge from the microstructure of this surface about the integrity of the sample mass. The images presented in Figure 6d–f show that the microstructure of GNC1-3 samples is more porous and cracks are visible in some places. This proves a better agglomeration of CAC particles with NS admixture in GNS1-3 pastes compared to the samples containing larger grains of NC.

### 3.4. XRD Analysis

**Figure 7:**
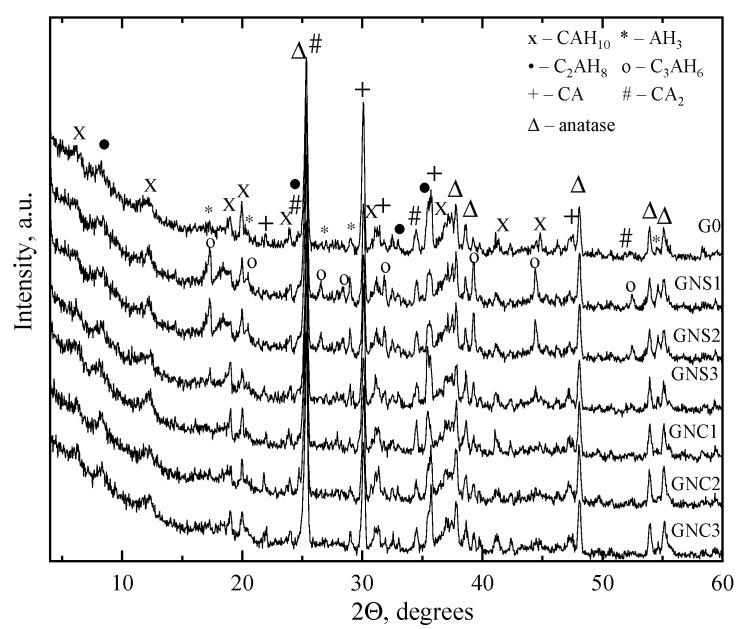
X-ray diffraction patterns of the cement pastes (with and without NC and NS) after 3 days of hardening (20 °C).

XRD analysis patterns of cement pastes with and without nano admixtures after 3 days of hardening are shown in Figure 7. The results of XRD analysis show that the following main crystalline products of CAC hydration were formed: CAH_10_ (d = 1.399; 0.722 nm), C_2_AH_8_ (d = 1.042; 0.522 nm) and AH_3_ (d = 0.484; 0.378 nm). On the XRD patterns, the peaks indicating the presence of unreacted cement minerals CA (d = 0.297; 0.192 nm) and CA_2_ (d = 0.349; 0.259 nm) are also visible. X-ray phase analysis (Figure 7, Table 3) showed that the same minerals were identified in control samples and in the samples with nano admixtures. However, in the case of pastes containing NS, the presence of C_3_AH_6_ (d = 0.513; 0.348 nm) was also observed.

Anatase was used as an internal standard; thus, it was possible to estimate the relative contents of the crystal components in the samples. The degree of hydration of the control CAC sample and the samples with addition of admixtures can be conditionally estimated from the intensities of the main peaks of the identified phases (Table 3). The relative evaluation of the content of crystalline phases according to the intensity of their main peaks after 3 days of hardening revealed that the total amount of hydrates in the samples (H*) with nano-silica was higher and the amount of not reacted cement minerals (N*) was lower compared to the results for the reference (Table 3).

Thus, the accelerating action of NS, visible in calorimetric results, was confirmed. In the case of NC-containing pastes, the estimated amounts of hydrated forms were slightly higher compared to the reference but lower compared to the samples with NS. Similarly, the amount of unhydrated phases is a bit lower than in the reference, but higher than in NS-admixtured pastes. It is seen that in NC-admixtured pastes, the early decelerating effect of NC, observed in calorimetric analysis results, weakens over time (after 72 h).

### 3.5. Infrared Spectroscopy

**Figure 8:**
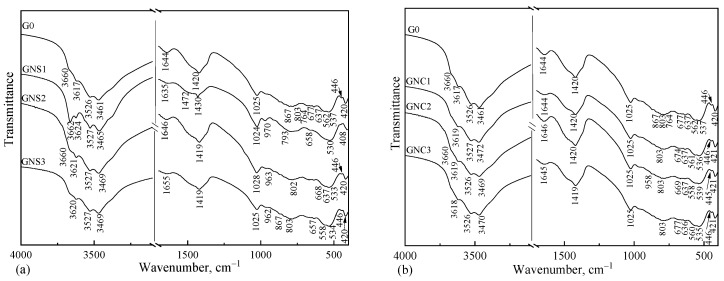
IR spectra of the hydrated CAC pastes without (G0) and with admixtures: NS (**a**) and NC (**b**).

The XRD method makes it possible to identify crystalline components of the samples. IR analysis was applied to supplement XRD results and to better characterize hydration products formed in the hardened CAC pastes [36]. The IR spectra of the hydrated CAC samples with and without the admixtures are shown in Figure 8a,b. In general, the spectra of the control hydrated sample (G0) and cement pastes prepared using different nano admixtures (GNS1-3 and GNC1-3) show similar absorption bands, which indicate the same hydration products in these samples.

XRD results of control sample (G0) make it possible to identify metastable hydrates, such as CAH_10_ and C_2_AH_8_, and the stable hydrate AH_3_. XRD results of the samples with NS showed metastable hydrates CAH_10_, C_2_AH_8_, and stable phases AH_3_ and C_3_AH_6_; in the sample with NC metastable hydrates, CAH_10_, C_2_AH_8,_ and the stable hydrate AH_3_ were identified. These compounds, as well as some phases that were not visible in XRD patterns, were identified in IR spectra.

A broad intense band in the absorption range of 3800–3000 cm^−1^ and a weak band at about 1645 cm^−1^ are due to the O–H groups in calcium aluminate hydrates and alumina gel. The latter band is related to H–O–H deformation vibrations. In the region of the higher wavenumbers, overlapping bands of stretching vibrations of water molecules as well as free OH groups bound in different hydrates can be observed. A few additional effects can be separated in this broad band. The IR spectra of CAH_10_ have a very broad and intense band in the 3550–3400 cm^−1^ region, with maxima located near 3500 cm^−1^. The effects at about 3620, 3525 and 3468 cm^−1^ are typical for gibbsite AH_3_ [37,38]. However, the peaks located at about 3618 and 3469 cm^−1^ probably are also due to the presence of C_2_AH_8_ [37,39]. The sharp intense band at about 3665 cm^−1^ is clearly visible only in the sample GNS1. The IR spectra of the pastes containing higher amounts of NS show only a shoulder in this region of wavenumbers, while the reference mixture and GNC1-3 do not exhibit this effect. This band results from the vibrations of OH-free groups in the cubic phase C_3_AH_6_ [37,38].

In the region of 1300–400 cm^−1^, a few overlapping bands of Al-O vibrations and some OH bending vibration bands can be identified. The absorption band at about 1025 and 970 cm^−1^ indicates the presence of gibbsite AH_3_ and is in agreement with the literature [37,38,39]. The bands at about 800, 658 and 530 cm^−1^, especially visible in the pastes containing NS, can also be assigned to gibbsite [38]. On the other hand, the above-described band at 1025 cm^−1^ may also derive from the presence of CAH_10_ [37] because the locations of these bands in the spectra are very similar and they overlap. The presence of CAH_10_ is also indicated by the bands at about 669 cm^−1^, a doublet close to 560–535 cm^−1^ [37], and the band at 408 cm^−1^ [38]. Other fundamental bands due to the stretching and bending vibrations of the Al–O in the AlO_6_ groups can be seen at about 802, 525 and 420 cm^−1^ [37,40,41]. These bands could overlap with the hexagonal phase absorption bands presented in the tested spectrum.

The third characteristic region in the IR spectrum of CAC hydration products is observed at 1300–1550 cm^−1^. In this range of wavenumbers, the stretching vibrations of ${\mathrm{CO}}_{3}^{2-}$ in carbonates were observed at about 1420 cm^−1^ (Figure 8). The band at 867 cm^−1^ can also be assigned as the band related to the presence of carbonates. Thus, it was found that both hexagonal and cubic hydration products are susceptible to carbonation and can react with atmospheric CO_2_.

The IR spectra analysis confirmed that both the hexagonal and the cubic hydration products coexist under the tested conditions. It was clearly shown that these results are in good agreement with the XRD analysis. It was also confirmed that NS affects hydration products while the influence of NC is inconsiderable. It can be seen that hydrated samples were also strongly affected by atmospheric CO_2_. Furthermore, the hydrated cement pastes containing NS were less sensitive to the carbonation process (with less depth of the absorption bands at about 1420 cm^−1^), which may be related to the different carbonation ability shown by hexagonal and cubic hydrates.

### 3.6. Thermal Analysis

DTG–TG is considered one of the most powerful tools in the investigation of the hydration of CAC pastes [29,31]. Similar to FTIR spectroscopy, thermal analysis can be considered as a method complementary to XRD, providing more detailed information about the composition of the samples. The interpretation of thermal analysis curves for hydrated CAC can be found in the literature, e.g., [18,38,39,42,43]. Although different temperature ranges of dehydration of specific products of CAC hydration can be found in the literature, the sequence of the appearance of these processes on TG/DTG/DTA curves remains the same [18,42].

**Figure 9:**
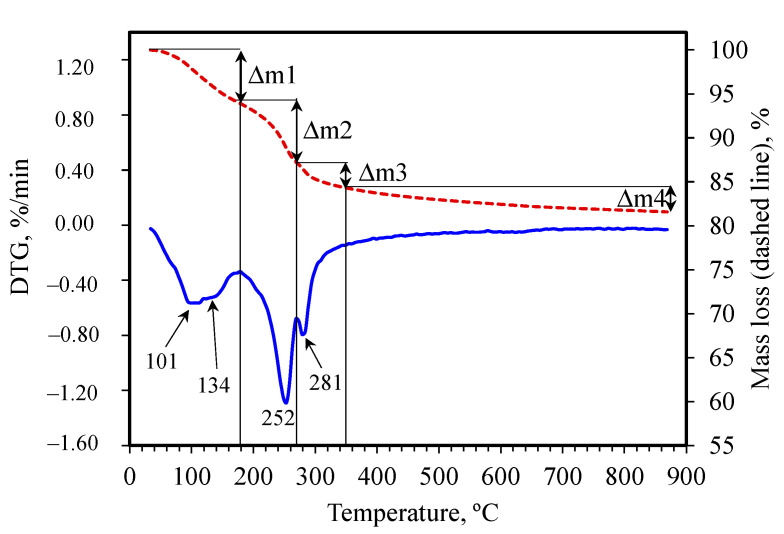
Exemplary TG and DTG curves for G0 sample and Δm1–Δm4—explanations in the text.

**Figure 10:**
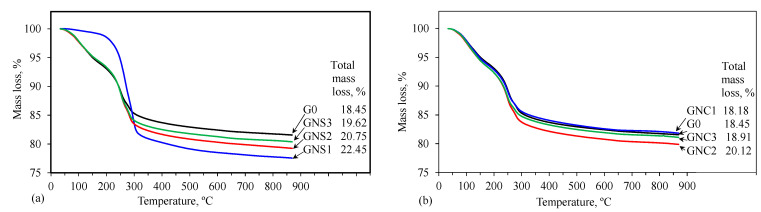
TG curves for cement pastes containing NS (**a**) and NC (**b**).

**Figure 11:**
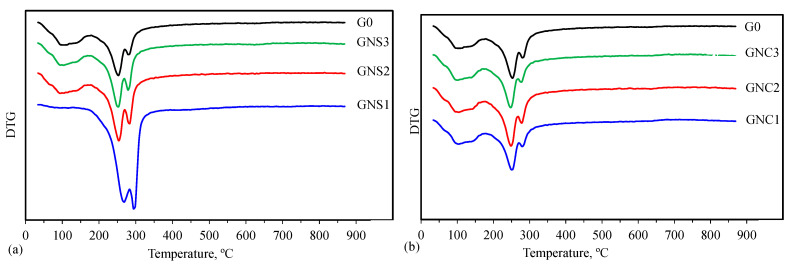
DTG curves for cement pastes containing NS (**a**) and NC (**b**).

**Figure 12:**
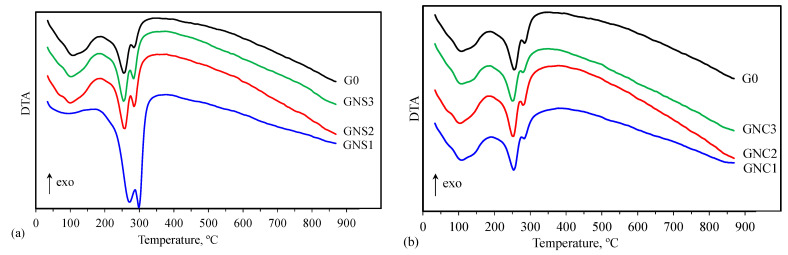
DTA curves for cement pastes containing NS (**a**) and NC (**b**).

TG, DTG and DTA results are depicted in Figure 9, Figure 10, Figure 11 and Figure 12.

A few overlapping endothermic mass losses in TG curves, linked with the peaks on DTG, can be specified at different temperature ranges [42,43]:

Δm1: from 50 °C to about 180 °C (extrema on DTG at about 100 °C and about 130 °C)—overlapping effects of dehydration of gel phases, such as AH_3_ gel and C-A-H gel (mainly at the lower temperatures), CAH_10_ hydrates (at about 100 °C, this dehydration follows the gel phase dehydration, the processes partially overlaps), and C_2_AH_8_ (mainly at the higher temperature range). 

Δm2: from about 180 to about 270 °C (the peak on DTG at 245–268 °C)—Dehydration of AH_3._

Δm3: from about 270 °C to 350 °C (DTG extremum at 276–296 °C)—Dehydration of C_3_AH_6._

Δm4: above 350 °C—Further dehydration, dehydroxylation and decarbonation.

The lack of the clear effect of CaCO_3_ decomposition (at about 700 °C) indicates that the carbonate bands observed in IR spectra are not connected with the presence of this compound. This confirms the formation of carboaluminate phases, the dehydration of which can be expected at the temperature close to the temperature of C_2_AH_8_ dehydration [42]. Thus, the dehydration of some carboaluminates also contributes to the first broad mass loss observed on the TG curve.

The TG analysis shows that the mass loss after heating the hardened cement sample up to 900 °C (Figure 10) is higher when the NS is used, especially when added at 0.05%. This can be explained by the larger amount of hydrates after the acceleration of cement hydration by NS (Figure 2d, Table 3).

The total mass losses (Figure 10) of the samples containing NC are similar to the results of the reference sample; only the paste with 0.1% of NC admixture (GNC2) shows about a 2% increase in mass loss compared to the control sample (G0). In the samples with NS admixture, an increase up to 4% is observed. It can be explained by the more intense formation of hydrates around particles of the nano admixture. Apparently, this increases the degree of cement hydration. Lower loss of weight can be the result of a smaller amount of hydrates. This data showing a lower degree of cement hydration (G0) is in good agreement with XRD results, where the greatest intensity of the non-hydrated phases is observed (Table 3).

The differences in the shapes of the thermal analysis curves indicate that the quantitative compositions of the investigated samples can be different. Taking into account that the products of CAC hydration depend on the temperature, one can expect that at 20 °C, i.e., the hardening temperature used in this research, CAH_10_ and a small amount of C_2_AH_8_ should be formed [6,31,44]. The thermal analysis results show that a small amount of regular hydrate C_3_AH_6_ is also created in the G0 sample. Similar observations regarding a few-day CAC hydration process at 20 °C were also made by other authors [17,18,20]. It is possible that despite the attempt to limit the self-heating effect of the sample, the temperature inside the paste could temporarily increase during the early periods of hydration. As a result, there were conditions for the formation of small amounts of cubic hydrate C_3_AH_6_ next to the main products, i.e., hexagonal hydrates CAH_10_ and C_2_AH_8_. The presence of the regular hydrate indicates that, despite the moderate hardening temperature and the short hydration time, conversion can take place to a minor extent. Thus, C_3_AH_6_ may be formed during hydration and conversion processes; however, a small amount of this hydrate and probably a low degree of its crystallinity are the reasons why it was not observed in XRD patterns for G0.

The shapes of TG, DTG and DTA curves registered for samples containing NS and NC show that their influence on the hydration products depends on the kind of admixture. The effect of NC is insignificant and all the curves are similar to those registered for the reference. On the contrary, for samples containing NS, this nano admixture favours formation of C_3_AH_6_. It is especially visible in the case of the sample containing the smallest amount of NS (GNS1), where the stable hydrates C_3_AH_6_ and AH_3_ are the main products of cement hydration. In the case of GNS2 and GNS3 samples, the amount of C_3_AH_6_ is clearly lower and it is decreasing with the increase of NS content. Thus, the results of thermal analysis confirm the IR and XRD findings.

Several factors may cause the above-discussed phenomenon. The presence of NS in the CAC paste causes the acceleration of hydration processes and more heat is released during the early periods after the addition of water. Thus, the temperature inside these mixtures could be higher compared to G0 and GNC1-3 samples. Elevated temperature and low w/c ratio can promote the formation of C_3_AH_6_ [17,45,46]. The presence of NS can improve the dissolution of CA (the main component of CAC). It was shown that the NS admixture, especially when it is used in the lowest amount, enhances the flowability of the mixture, which may cause better access of water to cement grains. The so-called effect of foreign ions also contributes to the improvement of the dissolution of CAC components. As a result, more Ca^2+^ ions appear in the solution and a calcium-rich phase C_3_AH_6_ can be more easily formed. This effect is especially visible in GNS1 paste. In the samples containing a larger amount of NS (GNS2 and GNS3), a more intensive adsorption of Ca^2+^ ions on the grains of the admixture can take place and this process can reduce the formation of C_3_AH_6_ [17] compared to GNS1 paste.

Results similar to those discussed in this paper were also obtained by Son et al. [17] for the samples containing nano-SiO_2_ and cured at 20°C for 7 days. However, it should be noted that the introduction of nano-SiO_2_ into CAC paste is often considered as a way to reduce the conversion of hexagonal hydrates to regular ones by creating a calcium alumino-silicate phase C_2_ASH_8_ [6,17,45]. The results indicate that this inhibition of conversion can take place only after exceeding a certain limit of nano-SiO_2_ content in the system, while a small amount of this admixture favours the formation of C_3_AH_6_ hydrates in early hydration stages. This different direction of the reaction is the reason why research into the mechanism of the action of nano-SiO_2_ in CAC paste, depending on the amount of this admixture, hydration time, temperature, and water to CAC ratio, should be continued.

**Figure 13:**
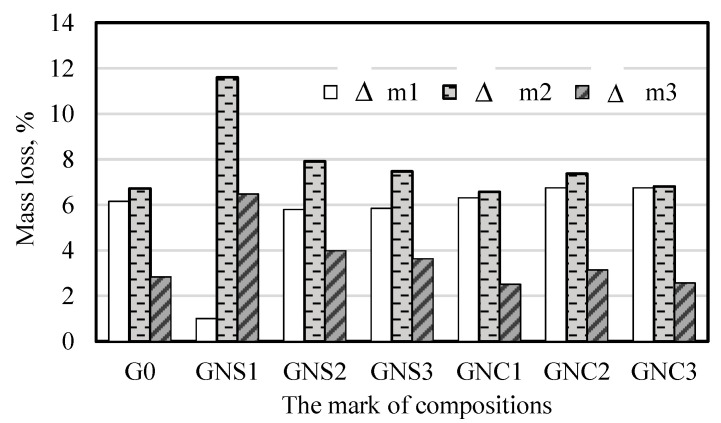
Mass loss at specific temperature ranges.

The quantification of CAC hydration products based on the thermal analysis curves is practically impossible because of overlapping effects of dehydration. In order to estimate the relative content of metastable and stable hydrates in the samples, the mass losses at the specific temperature ranges were presented in Figure 13. It can be seen that in the presence of NS, less water is bound in the metastable hexagonal hydrates (CAH_10_, C_2_AH_8_) compared to the reference, and more water is bound in the stable products (C_3_AH_6_, AH_3_). In the case of the sample with the lowest content of NS, C_3_AH_6_ and AH_3_ are the main products of hydration. The results obtained for the samples containing NC are similar to the reference, but a slight increase in the amount of water bound in the metastable products is visible. This confirms the previous conclusions drawn on the basis of X-ray diffraction results (Table 3).

### 3.7. Density, Compressive Strength and MIP Results

**Figure 14:**
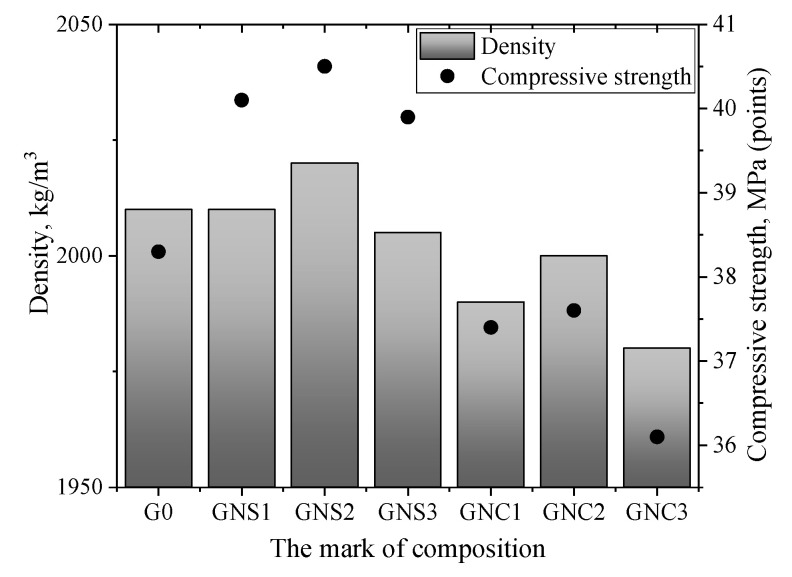
Results of compressive strength and density of cement pastes after 3 days of hardening.

Figure 14 presents the influence of NC and NS on the density and compressive strength of cement samples. The results demonstrate that the compressive strength increases with the addition of NS. We can see the formation of a denser structure after 3 days of hardening (Figure 14). The highest compressive strength at 3 days was obtained in the sample with NS added at 0.10 wt.%, where the compressive strength was found to be 6.5% higher than the strength of control samples. In spite of the stable hydrates mainly formed in the GNS1 paste, this blend shows a higher compressive strength value compared to the result for G0. This is because C_3_AH_6_ was created in the early hydration periods, not as a result of long-lasting conversion taking place in the hardened material. Thus, the compressive strength was not reduced in the GNS1 sample.

The decrease in strength was observed with the addition of NC compared to the reference. The sample containing 0.20 wt.% of NC (GNC3) showed the highest reduction in this mechanical property with the strength being 5.7% lower than the compressive strength of the control sample.

The comparison of the compressive strength values presented in Figure 14 (points) with the density values (columns) show the same nature of the variation of these characteristics. The nano admixture had a slight impact after hardening (from 10 to 30 kg/m^3^), whereas a more visible impact of nano admixtures (from 100 to 150 kg/m^3^) was observed after 3 days compared to the control sample.

**Figure 15:**
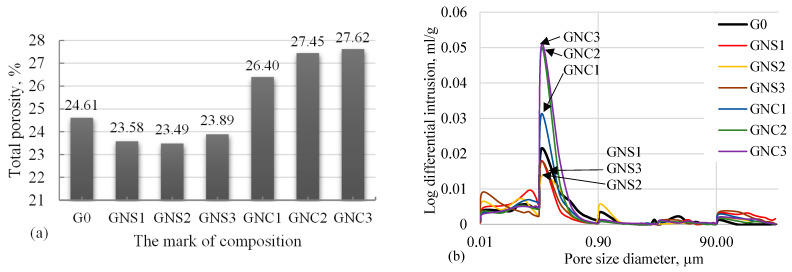
Results of total porosity (**a**) and pore size distribution (**b**) of CAC pastes.

The measurements of the total porosity and pore size distribution for characteristic CAC samples are presented in Figure 15a,b.

From the results given in Figure 15a, it is clear that the total porosity of the paste with NS admixture decreases. It should be noted, however, that the determination of porosity of hardened cement pastes requires prior drying of the samples. In this study, the samples were exposed to the temperature of 105 ± 5 °C, so it can be expected that their structure had changed. The conversion of hydrates can impact the porosity of CAC paste because the density of C_3_AH_6_ is higher than that of CAH_10_ or C_2_AH_8_ [47]. The measurements of the pore size distribution (Figure 15a,b) show that the pore size distribution in all cement samples follows a polymodal profile; the largest number of pores are ~0.1 µm in size. There are clear differences between the porosity of the samples with NS and NC. The pore volume (~0.1 µm size pores) was the lowest in the samples containing NS, whereas NC increase the pore volume by 3 times (Figure 15b). Little change in the pore volume of other pore sizes was observed in the samples with admixtures. The results given in Figure 15a show that the samples with NS also had lower total porosity and a denser microstructure. The authors of [48] explained the increase in porosity with the differences in the relative volumes of the phases. The more pronounced decrease in porosity in the samples of NS series can also be explained by the results of SEM analysis. The results of both analyses show that the samples with NS have more small pores (Figure 6b), and the samples with NC have more larger pores (Figure 6d–f).

However, the increased porosity of NC modified samples may be favourable in some cases for using this cement in refractory castable exposed to high temperatures. In such a case, greater porosity of the cement paste may be advantageous because the drying is less rapid and the risk of cracks in the material and explosive spalling, being a result of excessive increase in vapor pressure within the structure, is reduced [49,50]. Thus, there is a need to determine experimentally the optimal structure of the refractory castable containing nano admixtures. It seems to be beneficial to introduce a proper mixture of both nano materials, i.e., NC + NS, into the CAC paste. In this way, the synergistic effect of both nano materials could be observed and NC + NS containing binders may show advantageous properties. This requires experimental confirmation and such mixtures will be the subject of research in the future.

## 4. Conclusions

The main conclusion of this investigation is that nano admixtures, such as carbon nano-cones (NC) and nano-silica (NS), even added at very small amounts (0.05–0.2% by mass), impact the early hydration, structure formation, and physical and mechanical properties of CAC. The admixtures differ in chemical composition and the size of grains, thus their influence on cement paste depends mainly on the kind and amount of the material used. On the basis of the investigation results the following conclusions were formulated:

1. Based on the analysis of calorimetry results, it can be concluded that the admixtures change the kinetics of early cement hydration: NS accelerates the hydration processes and generates more heat, NC retard cement hydration and decrease the cumulative heat evolved. This is the effect of nucleating activity of NS grains which are significantly smaller compared to the grains of NC.
2. The presence of NS, especially used in the smallest amount, improves the flowability of the cement paste, while NC do not affect it significantly.
3. When NS was added to the CAC paste, after 3 days of hardening the cement paste showed better agglomeration of the particles, the microstructure was more densified, and small-size hydrates were formed. The samples containing NS also exhibited lower porosity. In the case of the pastes with NC, the microstructure was more porous with cracks in some places, and the total porosity was higher.
4. In general, the same kinds of hydration products were created in all CAC pastes tested, although the quantitative composition was different. NC practically had no influence on the hydration products formed compared to the results of the reference paste, while the presence of NS changed the hydration mechanism.
5. In the case of NS, more hydrates C_3_AH_6_ and AH_3_ were formed compared to the reference paste and the pastes containing NC. This is especially visible in the pastes with the lowest content of NS admixture. This is probably due to the complex effect of self-heating of the paste, low w/c ratio, an improvement of dissolution of cement grains, and acceleration of its hydration.
6. The compressive strength of cement pastes after 3 days of hydration was higher in NS-admixtured pastes, and lower for NC-admixtured samples, compared to the results of the reference sample.

In summary, the properties of CAC samples containing NS are promising due to the possibility to use them in refractory castables. The main advantages of such mixtures after 3 days of hydration at 20 °C are small sized and poorly crystallized hydration products, and a compact hardened microstructure. NS admixture prevents the conversion of hydrates, promotes immediate formation of stable and denser hydrates (C_3_AH_6_ and AH_3_) at early hydration periods, and makes it possible to control morphology; therefore, it improves the quality of refractory castable. The mechanism of the influence of very small amounts of NS on CAC hydration is interesting and atypical, and requires further in-depth investigation.

## Figures and Tables

**Table 1:** Composition and properties of the CAC used.

Component	Typical Values (%)	Main Phases
Al_2_O_3_	71.1	CA:CaO·Al_2_O_3_, CA_2_:CaO·2Al_2_O_3_, Additional phases: C_12_A_7_:12CaO·7Al_2_O_3_, A:α-Al_2_O_3_
CaO	28.0	
SiO_2_	<0.5	
Fe_2_O_3_	<0.3	
Na_2_O + K_2_O	<0.5	

**Table 2:** Compositions (mass %) of the samples tested.

The Mark of Composition	G70 (%)	NS (%)	NC (%)	W/C
G0	100	-	-	0.35
GNS1	100	0.05	-	0.35
GNS2	100	0.1	-	0.35
GNS3	100	0.2	-	0.35
GNC1	100	-	0.05	0.35
GNC2	100	-	0.1	0.35
GNC3	100	-	0.2	0.35

**Table 3:** The intensity of the main peaks of minerals.

The Mark of Compositions	CAH_10_	C_2_AH_8_	AH_3_	C_3_AH_6_	H*	CA_2_	CA	N*	Anatase
G0	117	64	101	-	282	497	401	898	450
GNS1	97	54	139	231	521	525	250	775	450
GNS2	114	64	141	230	549	519	283	802	450
GNS3	114	53	105	138	410	482	335	817	450
GNC1	125	67	108	-	300	530	280	810	450
GNC2	114	69	106	-	289	514	319	833	450
GNC3	107	72	104	-	283	553	303	856	450

H*—Total amount of hydrates; N*—Total amount of non-hydrated phases (both estimated as a sum of intensities of main peaks).

## References

[1] Heikal M., Radwan M.M., Morsy M.S. (2004). Influence of curing temperature on the physicomechanical, characteristics of calcium aluminate cement with air-cooled slag or water-cooled slag. Ceram. Sil..

[2] Roy J., Chandra S., Maitra S. (2019). Nanotechnology in castable refractory. Ceram. Int..

[3] Garbers-Craig A.M. (2008). How cool are refractory materials?. J. S. Afr. Inst. Min. Metall..

[4] Shiri S., Abbasi M.H., Monshi A., Karimzadeh F. (2014). A study on mechanical and physical properties of monocalcium aluminate cement reinforced with nano-SiO_2_ particles. Compos. Part B-Eng..

[5] Li H., Hui-gang Xiao H.G., Ou J.P. (2004). A study on mechanical and pressure-sensitive properties of cement mortar with nanophase materials. Cem. Concr. Res..

[6] Abd El-Hamid H.K., Radwan M.M. (2019). Influence of nano-silica additions on hydration characteristics and cytotoxicity of calcium aluminate as biomaterial. Heliyon.

[7] Guo C., Wang E., Hou X., Chen J., Zhang W., Ye J., Qin S. (2020). Characterization and mechanism of early hydration of calcium aluminate cement with anatase-TiO_2_ nanospheres additive. Constr. Build. Mater..

[8] Rodger S.A., Double D.D. (1984). The chemistry of hydration of high alumina cement in the presence of accelerating and retarding admixtures. Cem. Concr. Res..

[9] Du S., Wu J., AlShareedah O., Shi X. (2019). Nanotechnology in Cement-Based Materials: A Review of Durability, Modeling, and Advanced Characterization. Nanomaterials.

[10] Zhang M.H., Islam J. (2012). Use of nano-silica to reduce setting time and increase early strength of concretes with high volumes of fly ash or slag. Constr. Build. Mater..

[11] Kawashima S., Hou P., Corr D.J., Shah S.P. (2013). Modification of cement-based materials with nanoparticles. Cem. Concr. Compos..

[12] Wang L., Zheng D., Zhang S., Cui H., Li D. (2016). Effect of nano-SiO_2_ on the hydration and microstructure of portland cement. Nanomaterials.

[13] Hui Li H., Xiao H.G., Yuan J., Ou J. (2004). Microstructure of cement mortar with nano-particles. Compos. Part B-Eng..

[14] Singh L.P., Karade S.R., Bhattacharyya S.K., Yousuf M.M., Ahalawat S. (2013). Beneficial role of nanosilica in cement based materials—A review. Constr. Build. Mater..

[15] Malaiškienė J., Costa C., Banevičienė V., Antonovič V., Vaičienė M. (2021). The effect of nano SiO_2_ and spent fluid catalytic cracking catalyst on cement hydration and physical mechanical properties. Constr. Build. Mater..

[16] Wang F., Chen P., Li X.C., Zhu B. (2018). Effect of Colloidal silica on the hydration behavior of calcium aluminate cement. Materials.

[17] Son H.M., Park S.M., Jang J.G., Lee H.K. (2018). Effect of nano-silica on hydration and conversion of calcium aluminate cement. Constr. Build. Mater..

[18] Chavda M.A., Bernal S.A., Apperley D.C., Kinoshita H., Provis J.L. (2015). Identification of the hydrate gel phases present in phosphate-modified calcium aluminate binders. Cem. Concr. Res..

[19] Shinmei T., Ohkawa M., Borovsky A., Iiyama M., Parr C. (2013). The Formation of Stratlingite in Calcium Aluminate Containing Castable Systems. Refract. Worldforum 5.

[20] Du M., Jing H., Gao Y., Su H., Fang H. (2020). Carbon nanomaterials enhanced cement-based composites: Advances and challenges. Nanotechnol. Rev..

[21] Kudžma A. (2020). Effect Of Graphene Oxide on the Hydration, Structure and Properties of Cementitious Materials. Ph.D. Thesis.

[22] Liu T.T., Cao M.Q., Fanga Y.S., Zhu Y.H., Cao M.S. (2022). Green building materials lit up by electromagnetic absorption function:A review. J. Mater. Sci. Technol..

[23] Chen J., Liang C., Li B., Wang E., Li G., Hou X. (2018). The effect of nano-γAl_2_O_3_ additive on early hydration of calcium aluminate cement. Constr. Build. Mater..

[24] Claramunt J., Ventura H., Filho R.D.T., Ardanuy M. (2019). Effect of nanocelluloses on the microstructure and mechanical performance of CAC cementitious matrices. Cem. Concr. Res..

[25] Sanchez F., Zhang L., Ince C., Bittnar Z., Bartos P.J.M., Nemecek J., Smilauer V., Zeman J. (2009). Multi-scale performance and durability of carbon nanofiber/cement composites. Nanotechnology Construction 3.

[26] Rambo D.A.S., Ukrainczyk N., Silva F.A., Koenders E., Filho R.D.T., Gomes O.F.M. (2019). Calcium-aluminate mortars at high temperatures: Overcoming adverse conversion effects using clinker aggregates. Cem. Concr. Compos..

[27] Antonovič V., Pundienė I., Stonys R., Čėsnienė J., Kerienė J. (2010). A review of the possible applications of nanotechnology in refractory concrete. J. C. Eng. Manag..

[28] Sakai E., Sugiyama T., Saito T., Daimon M. (2010). Mechanical properties and micro-structures of calcium aluminate based ultra-high strength cement. Cem. Concr. Res..

[29] Antonovič V., Aleknevičius M., Keriene J., Pundienė I., Stonys R. (2012). Investigating the hydration of deflocculant calcium aluminate cement-based binder with catalyst waste. J. Therm. Anal. Calorim..

[30] Collier N.C., Sharp J.H., Milestone N.B., Hill J., Godfrey I.H. (2008). The influence of water removal techniques on the composition and microstructure of hardened cement pastes. Cem. Concr. Res..

[31] Pacewska B., Nowacka M., Antonovic V., Aleknevicius M. (2012). Investigation of early hydration of high aluminate cement-based binder at different ambient temperatures. J. Therm. Anal. Calorim..

[32] Pacewska B., Wilinska I., Bukowska M. (2009). Calorimetric investigations of the influence of waste aluminosilicate on the hydration of different cements. J. Therm. Anal. Calorim..

[33] Puerta-Falla G., Kumar A., Gomez-Zamorano L., Bauchy M., Neithalath N., Sant G. (2015). The influence of filler type and surface area on the hydration rates of calcium aluminate cement. Constr. Build. Mater..

[34] Juan Wang J., Xu Y., Wu X., Zhang P., Hu S. (2020). Advances of graphene- and graphene oxide-modified cementitious materials. Nanotechnol. Rev..

[35] Rashad A.M. (2013). Effects of ZnO_2_, ZrO_2_, Cu_2_O_3_, CuO, CaCO_3_, SF, FA, cement and geothermal silica waste nanoparticles on properties of cementitious materials–a short guide for Civil Engineer. Constr. Build. Mater..

[36] Torrens-Martın D., Fernandez-Carrasco L., Blanco-Varela M.T. (2013). Conduction calorimetric studies of ternary binders based on Portland cement, calcium aluminate cement and calcium sulphate. J. Therm. Anal. Calorim..

[37] Fernandez-Carrasco L., Torrens-Martın D., Morales L.M., Martinez-Ramirez S., Theophanides T. (2012). Infrared spectroscopy in the analysis of building and construction materials. Infrared Spectroscopy.

[38] Hidalgo A., García J.L., Cruz Alonso M., Fernández L., Andrade C. (2009). Microstructure development in mixes of calcium aluminate cement with silica fume or fly ash. J. Therm. Anal. Calorim..

[39] Madej D., Szczerba J. (2015). Study of the hydration of calcium zirconium aluminate (Ca7ZrAl6O18) blended with reactive alumina by calorimetry, thermogravimetry and other methods. J. Therm. Anal. Calorim..

[40] Tarte P. (1967). Infra-red spectra of inorganic aluminates and characteristic vibrational frequencies of AlO_4_ tetrahedra and AlO_6_ octahedra. Spectrochim Acta A Mol Spectrosc..

[41] Fernandez-Carrasco L., Vazquez T. (1996). Aplicacion de la espectroscopıa infrarroja al studio de cement aluminoso. Mater. Constr..

[42] Bushnell-Watson S.M., Sharp J.H. (1992). The application of thermal analysis to the hydration and conversion Reactions of calcium aluminate cements. Mater. Constr..

[43] Ukrainczyk N., Matusovic T., Kurajica S., Zimmermann B., Sipusic J. (2007). Dehydration of layered double hydroxide–C_2_AH_8_. Thermochim. Acta..

[44] Scrivener K.L., Cabiron J.L., Letourneux R. (1999). High-performance concretes from calcium aluminate cements. Cem. Concr. Res..

[45] Kim H., Son H.M., Lee H.K. (2021). Review on recent advances in securing the long-term durability of calcium aluminate cement (CAC)-based systems. Funct. Compos. Struct..

[46] Mostafa N.Y., Zaki Z.I., Elkader O.H.A. (2012). Chemical activation of calcium aluminate cement composites cured at elevated temperature. Cem. Concr. Compos..

[47] Gosselin C. (2009). Microstructural Development of Calcium Aluminate Cement Based Systems with and without Supplementary Cementitious Materials. Ph.D. Thesis.

[48] Ding J., Fu Y., Beaudoin J.J. (1996). Study of hydration mechanisms in the high alumina cement–sodium silicate system. Cem. Concr. Res..

[49] Luz A.P., Moreira M.H., Wöhrmeyer C., Parr C., Pandolfelli V.C. (2019). Drying behavior optimization of dense refractory castables by adding a permeability enhancing active compound. Ceram. Int..

[50] Nouri-Khezrabad N., Braulio M.A.L., Pandolfelli V.C., Golestani-Fard F., Rezaie H.R. (2013). Nano-bonded refractory castables. Ceram. Int..

